# Fat Body—Multifunctional Insect Tissue

**DOI:** 10.3390/insects12060547

**Published:** 2021-06-11

**Authors:** Patrycja Skowronek, Łukasz Wójcik, Aneta Strachecka

**Affiliations:** Department of Zoology and Animal Ecology, University of Life Sciences in Lublin, Akademicka 13, 20-950 Lublin, Poland; lukasz7wojcik@gmail.com (Ł.W.); aneta.strachecka@up.lublin.pl (A.S.)

**Keywords:** insect immunology, trophocytes, humoral immunity, insect metabolism, metamorphosis

## Abstract

**Simple Summary:**

Efficient and proper functioning of processes within living organisms play key roles in times of climate change and strong human pressure. In insects, the most abundant group of organisms, many important changes occur within their tissues, including the fat body, which plays a key role in the development of insects. Fat body cells undergo numerous metabolic changes in basic energy compounds (i.e., lipids, carbohydrates, and proteins), enabling them to move and nourish themselves. In addition to metabolism, the fat body is involved in the development of insects by determining the time an individual becomes an adult, and creates humoral immunity via the synthesis of bactericidal proteins and polypeptides. As an important tissue that integrates all signals from the body, the processes taking place in the fat body have an impact on the functioning of the entire body.

**Abstract:**

The biodiversity of useful organisms, e.g., insects, decreases due to many environmental factors and increasing anthropopressure. Multifunctional tissues, such as the fat body, are key elements in the proper functioning of invertebrate organisms and resistance factors. The fat body is the center of metabolism, integrating signals, controlling molting and metamorphosis, and synthesizing hormones that control the functioning of the whole body and the synthesis of immune system proteins. In fat body cells, lipids, carbohydrates and proteins are the substrates and products of many pathways that can be used for energy production, accumulate as reserves, and mobilize at the appropriate stage of life (diapause, metamorphosis, flight), determining the survival of an individual. The fat body is the main tissue responsible for innate and acquired humoral immunity. The tissue produces bactericidal proteins and polypeptides, i.e., lysozyme. The fat body is also important in the early stages of an insect’s life due to the production of vitellogenin, the yolk protein needed for the development of oocytes. Although a lot of information is available on its structure and biochemistry, the fat body is an interesting research topic on which much is still to be discovered.

## 1. Introduction

Insects are one of the most diverse and numerous groups of organisms. Due to their biodiversity, insects occupy all ecological niches, which benefit from their irreplaceable services [1]. The unique biology and behavior of some insects have been noticed and described [2,3]. Insects appear in human life as producers of health-promoting food (*Apis mellifera*), pollinators (Hymenoptera and Lepidoptera), producers of exclusive materials (*Bombyx mori*), model organisms (*Galleria mellonella*), and high-protein food (crickets, locusts, and mealworms) [4,5,6,7,8,9]. Utility insects are useful, but also cause many problems related to their breeding and use, including the loss of resistance, which can be due to many factors. Growing environmental pollution, parasites, pathogens, and selective breeding adversely affect their bodies. The complete disappearance of the natural populations of some species (*B. mori*) is a great danger to the environment [10]. In response to these worrying developments, research is being carried out on key tissues to determine their functioning and prevent premature degradation due to negative factors. Despite their species richness, insects have multifunctional tissues common to most species [5,11]. In this review, we focus on the fat body, which is multifunctional and the first biologically active protective barrier. The fat body has a fairly diverse structure with common elements. It is located in many areas of an insect’s body, including under the body surface and surrounding organs. The main tasks of the fat body are the synthesis, transport, accumulation, and release of the main organic compounds, i.e., proteins, lipids, and carbohydrates [5]. Metabolism inside the fat body tissue occurs in very close contact with other major tissues, such as the hemolymph (i.e., “insect blood”), which is reflected in the functioning of the whole organism [12,13]. It is crucial to understand the functioning and processes that occur in the fat body due to its role in creating immunity, and in the proper growth and metamorphosis of insects from larvae to imagoes (i.e., maximum use of the organism’s potential and resources) [11,14,15,16].

## 2. Morphology and Anatomy of the Fat Body

The fat body is made up of five main types of cells, which vary in composition, size, and function during the different stages of growth [11,17]. The morphology of the tissue is the same within a given species, but there may be some differences between species (e.g., different arrangement of cells) [17,18].

The most numerous and basic cells are trophocytes, formerly known as adipocytes, but the name was inappropriate because it failed to take into account the multifunctionality of these cells. Due to its location and functions (Section 3—“Differentiation of the Fat Body”, page 5), the fat body has various physiologies. Usually, the cells of the fat body form organized, compact, and thin structures. This form can be found in the “subcutaneous” fat body located just below the insect’s body surface. The close arrangement of cells allows for very good contact and transmission of signals between the complexes of the fat body [18]. Fat tissue in the abdominal cavity, thorax area, or head surrounds the organs located there and may form less-regular structures [19]. In addition, most of the flaps of the tissue come into contact with the hemolymph to integrate the signals from all corners of the entire body [20]. At individual stages, growth of this tissue is observed in insects, and the largest cells of the fat body are found in the oldest larvae [18,21].

### 2.1. Trophocytes

Trophocytes are polymorphic cells of mesodermal origin. These cells predominantly take care of the storage, secretion, and detoxification of the organic substances present in the insect body. The entire cell is covered with a thin, protective film that hides other organelles underneath [11,18].

The central cell consists of an irregular nucleus surrounded by a cytoplasmic ring, storage structures (granules and drops of accumulated lipids), and protein compounds embedded in the cytosol, as well as large vesicular bodies and vacuoles. Other regularly occurring structures in the cell include round or long mitochondria, the Golgi apparatus (occupying more or less of the surface depending on the life stage), or the endoplasmic reticulum, which forms a peripheral labyrinth of channels limited by varying extents of membranes [18].

Cells can vary in size due to the accumulation of nutrients and the swelling of the vacuoles therein (Photo 1). Vacuoles usually grow in size some time before the individual transitions between the developmental stages of larva to imago. Before metamorphosis, digestive vacuoles shrink, which may be related to the overall reduction in body fat (Section 3—“Differentiation of the Fat Body”, page 5). There are the following four types of major vacuoles in the cells of the fat body: digestive, storage, condensation, and superficial. Digestive vacuoles (autophagous, heterophagous) have the ability to process and digest, releasing spare substances into the body during energy expenditure or diapause. Such vacuoles also contain remains of digested organelles during collaboration with lysosomes (Section 9—“Role in Immunity”, page 17). Storage vacuoles are responsible for the storage of spare substances in the form of tyrosine, urates, proteins, lipid drops, and sometimes symbiotes present in glycogen deposits. Structures containing glycogen appear as membranous structures. The glycogen content is not seasonal. Condensation vacuoles are related to the Golgi apparatus and lysosomes, and often contain proteins. Superficial vacuoles are formed by the fusion of vesicles (provacuoles) [17,18]. The changing appearance of organelles, due to the accumulation of nutrients such as lipids, is visible in the cytoplasm, as well as the appearance of the endoplasmic reticulum and the size of the lipid droplets. Insects consuming high-fat meals are characterized by a slightly elongated mitochondrion and a stronger development of the rough endoplasmic reticulum compared to insects tested in the fasting state [22].

The cytoplasm also shows pinocytosis-derived protein granules that look like coated vesicles. Although they appear in the image of the fat body, their task is to selectively absorb proteins from the hemolymph. The selectivity is probably related to the specific connections between the membrane receptors already produced during pinocytosis. Selectively absorbed proteins are species-specific. In addition to these proteins taken from the hemolymph, other proteins are synthesized in fat body cells. Due to the contact of the fat body with the hemolymph, protein exchange takes place between them, which integrates the entire body. The existing Golgi-like granules are also formed with secretions from cellular synthesis [17,18].

In addition to differences in the structures of trophocytes, the number of these cells varies. The number of trophocytes is lower in male insects than in females, with a higher production of vitellogenin (Section 9—“Role in Immunity”, page 15). Such changes are noticeable between the cast registers in insects that have them. A higher number of cells are also present during molting (mesoderm differentiation) [11,17,18].

### 2.2. Oenocytes

**Figure 1:**
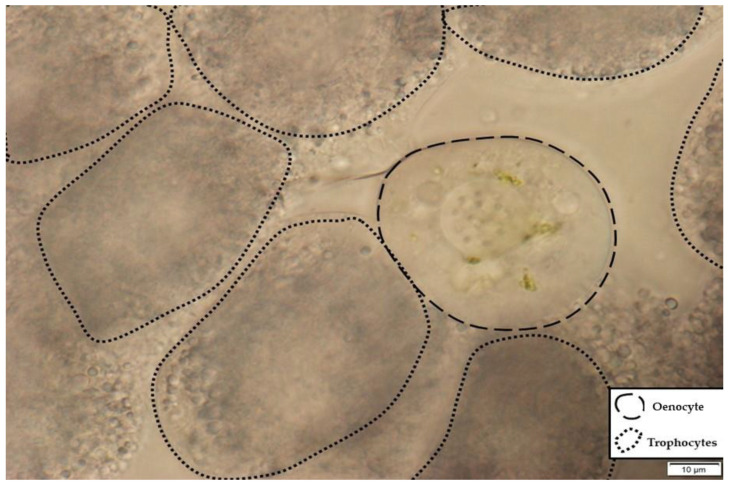
Subcuticular fat body cells under a light microscope: trophocytes and oenocyte.

Oenocytes are circular or oval cells associated with the epidermal layer of the cuticle, together or separately with the mainly parietal fat body. Oenocytes have a centrally placed nucleus, mitochondria, smooth endoplasmic reticulum, and vacuoles containing drops and granules of lipids, proteins, and glycogen. They are distributed throughout the body or occur in small groups (e.g., around the spiracles). They are often darker in color than trophocytes (brown/yellow/amber) (Figure 1) and are the second most abundant type of fat body cell, despite their low abundance compared to trophocytes. They have the ability to synthesize the carbohydrates transported between the hemolymph and the fat body. In some species (especially Diptera insects, e.g., *Chironomus thummi*), oenocytes also contain hemoglobin [11,23,24].

### 2.3. Mycetocytes

Mycetocytes are cells that contain mainly symbiotic prokaryotic microorganisms. They live in permanent symbiosis with the insect at a certain number, which can be confirmed by research conducted on *Periplaneta american* cockroaches. In this insect species, several representatives of the symbiotes inhabiting these cells have been found, showing unchanging activity until the host dies. As with other cell types, granules of fat and glycogen are observed in mycetocytes, often with a reduced amount of cytoplasm. Mycetocytes are found in organisms that eat poor-quality, unbalanced food, synthesizing some nutrients, i.e., amino acids or B vitamins [11,25].

### 2.4. Chromatocytes

Chromatocytes are flat cells containing a central nucleus and a transparent cuticle, located in the thinnest layers of the fat body. They accumulate fats that the insect uses during its metamorphosis. These cells are present in some aquatic insects [11].

### 2.5. Urocytes

Urocytes are characterized by the presence of a reduced endoplasmic reticulum and a vacuole with accumulated urate granules. Urate comes from the metabolism of nucleic acids or protein degradation. The main purpose of these cells is to store the urate granules [11,18].

### 2.6. Microbiota of the Fat Body

During research on the fat body microbiota of *Nilaparvata lugens* (brown planthopper), the presence of microorganisms was noted, specifically belonging to *Firmicutes*, *Proteobacteria*, *Actinobacteria*, *Bacteroidetes*, *Fusobacteria*, *Cyanobacteria*, *Chlamydiae*, *Deferribacteres*, and *Saccharibacteria* clusters. The largest percentage (up to 99% of clusters) was *Firmicutes andProteobacteria*. The types of microbes in the fat body belong to *Bacillus*, *Arsenophonus*, *Lactococcus*, *Asaia*, *Enterococcus*, *Streptococcus*, and *Carnobacterium* [26].

## 3. Differentiation of the Fat Body

**Figure 2:**
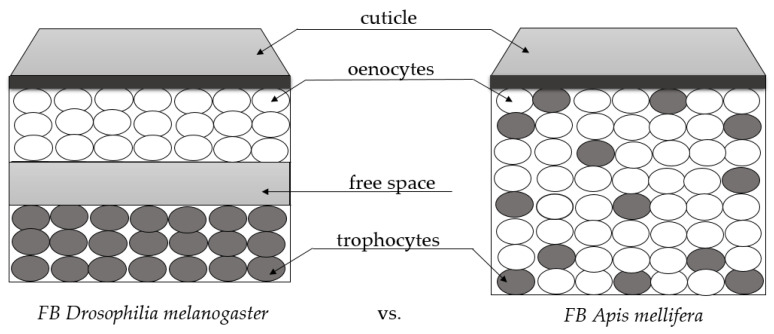
Differences in the structure of the subcuticular fat body (FB) between the fly (*D. melanogaster*) and bee (*A. mellifera*).

Fat body research presents scientists with many challenges because it is related to the differentiated structure of cells and tissues (structural and regional) depending on the type, species, stage of development, sex, caste, physiological state, environmental conditions, and even the location of the tissue in the insect organism. This creates many problems for the analysis of the same tissue, as the material must be collected from the right place under the right conditions for the results to be reliable [27]. In many species, we see the fat body as a tissue in which different types of cells are mixed together. However, new research shows that the fat body may be composed of several layers, with a given layer dedicated to only one type of cell (e.g., in *Drosophila melanogaster*). In flies, the subcuticular fat body is composed of several layers separated by free spaces. The layer contains only one type of trophocyte or oenocyte, which facilitates their preparation (Figure 2) [28]. In other insects (including the less primitive ones), such as the honey bee, the mixing of cells is observed at the level of one layer.

### 3.1. Histotypes and Regional and Structural Differences

Due to the regional differentiation in insects, one organism may contain various cells and tissues of the fat body, such as axial, perivascular (visceral), and subcutaneous (peripheral). As described previously, the fat body most often occurs in the form of thin sheets that are in contact with the hemolymph. Such an arrangement of cells has been noted in part of the front and rear axles in flies. Another form of cell arrangement is in the center of the insect body cavities. The cells of the fat body are arranged there in prominent flaps surrounding the intestine, called perivascular or visceral fat bodies. The lobe structure is also located under the armor layer directly related to the epidermis. These cells are often separated from the internal cavities of the body by the muscles. This localization of the fat body is called peripheral or subcutaneous [27].

Regional and structural differentiation influences the appearance of cells in microscopic images. Due to this differentiation, several cell histotypes have been described. The differences between the histotypes are observed on the basis of the color of the tissues, as follows: white, thick patches are found in most insects (e.g., earworm); brown (e.g., Indian moth); colored/mixed in Lepidoptera insects, yellow and even blue in color. The color depends on the structure, development and accumulation of proteins within a given tissue. The content of pigments, such as ammochrome and pteridines, which are precursors of eye pigment in the fly, or the structure of glycogen in the Colorado beetle, may also affect the tissue image. The content of these pigments in various parts of the fat body indicates their broad role depending on where they are located [27]. Xanthomatins (eye pigments), which act as a filter, increasing the contrast, accumulate in the front three sections of the thorax. This location allows these dyes to be applied quickly to a closely located eye [29,30]. In contrast, pteridines (derived from guanine) accumulate in the last three segments of the abdomen [27].

### 3.2. Metamorphosis of the Fat Body

In insects, during their first phases of life, fat cells develop from embryonic progenitor cells. They continue to multiply through the mitotic process, and then differentiate, as we mentioned above. The resulting fat body cells constantly multiply by endoreplication [31,32]. The growth of insects is possible due to the processes of molting and metamorphosis.

The transformation of insects (metamorphosis) into the adult form may occur through the following two processes: by complete histolysis of the fat tissue cells in the larva and the production of new tissues for the imago (in holometabolic insects), or by remodeling previous cells into adult cells [21,27]. The changes and development (body remodeling) are induced by the hormone 20-hydroxyecdysone (20E). Molting and metamorphosis in insects occurs due to the activation of the transcriptional cascade as a result of the stimulation of the nuclear receptor ecdysone receptor/ultraspiracle protein (EcR/USP) by the action of 20E [33]. The hormone is usually activated by factors similar to starvation conditions.

The first stage of histolysis is the dissociation of the fat body cells [27]. The following wo metalloproteinases are involved in this process: Mmps 1 is responsible for the cleavage of adjacent cells, whereas Mmps 2 degrades the basal membrane that covers the fat body tissue. Mmps 1–2 are also regulated by the following hormones: E20 and juvenile hormone (JH). After the action of metalloproteinase, we obtain free spherical cells floating in the hemocele [34,35]. In Lepidoptera insects (silkworms), cathepsin is activated by 20E and also takes part in dissociation. Despite the presence of cathepsins in Diptera, it is not a key protease in metamorphosis [36,37]. After the tissue breaks down into smaller structures, the hemolymph-derived hemocytes inhibit further separation [27].

Also, 20E is involved in the activation of programmed cell death (PCD). After proper dissociation, histolysis occurs via cell apoptosis. Them, 20E activates the caspase activity cascade (CAC). CAC is involved in the destruction of cell structures and organelles. After dissociation, the separated fat body cells are reached by cysteine proteinase, which digests the membrane and causes the cells to break down [38]. CAC, together with the action of autophagy-related protein (ATG) 5–6, leads to cell apoptosis [39]. Apoptosis can also be activated as an adaptation to adverse environmental conditions. The previously mentioned hunger (food signals) activates the enzyme 5′ adenosine monophosphate-activated protein kinase (AMPK) and the transducer of regulated CREB activity 1 (TORC1). These molecules regulate ATG10–ATG13, which in turn initiates the activation of the autophagosome. At the same time, in cooperation with 20E, EcR/USP and ecdysone-induced protein 93 (E93, present e.g., in cockroaches), in the presence of gene regulator Myc, block the IIS-TORC1 pathway, which also contributes to autophagy activation related to the regulation of nutritional signals [40,41,42,43,44]. Approximately 2200 cells are completely histolyzed [27].

**Scheme 1:**
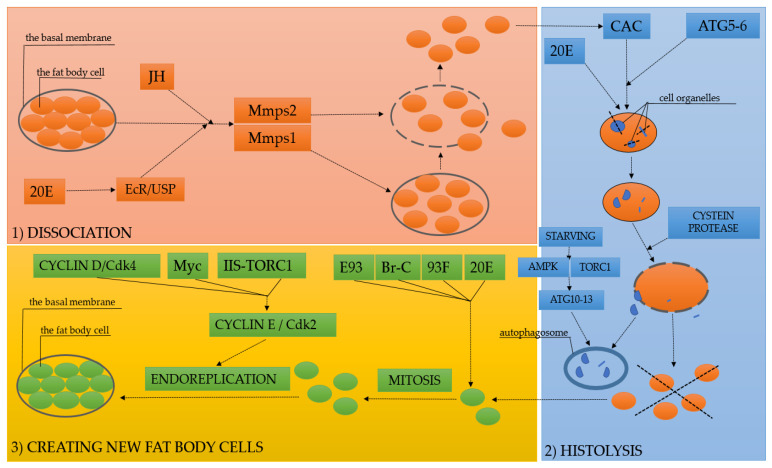
The process of metamorphosis of the fat body of insects. JH—juvenile hormone; 20E—20-hydroxyecdysone; EcR/USP—ecdysone receptor/ultraspiracle protein; Mmps 1–2—metallo-proteinases; CAC—caspase activity cascade; ATG5–6—autophagy-related proteins 5–6; AMPK—5′ adenosine monophosphate-activated protein kinase; TORC1—transducer of regulated CREB activity 1; ATG10–13—autophagy-related proteins 10–13; 93F—proper name of protein; Br-C—Broad-Complex gene; E93—ecdysone; IIS-TORC1—connection between IIS—Sensors insulin/IGF signaling and TORC1 signaling; Myc—proper name of gene regulator; Ckd2—cyclin-dependent kinase 2; Ckd4—cyclin-dependent kinase 4.

When new tissues are produced, the fat body develops from the fat body remaining after histolysis. The production of new cells is induced by the activity of 20E, the 93F protein, ecdysone (E93) and the Broad-Complex (Br-C) gene [33,38,45]. Cells undergo mitosis, reaching a cell number of roughly 2000. These 2000 cells are then undergo endoreplication by cyclin E/cyclin-dependent kinase (Ckd2). Cyclin E/Ckd2 is activated by the activity of Myc, the IIS-TORC1 pathway, and cyclin D/Cdk4 (Scheme 1) [46,47]. Three days after the insect enters this stage, the number of cells increases to approximately 18,000 [48]. The end of the process of new tissue formation occurs by the suppression of the expression of E93 and Br-C by the activity of Krüppel homolog (Kr-h1). Kr-h1 expression is influenced by JH in association with the Met receptor [49]. The resulting cells constitute the new fat body [27,49].

Remodeling is the transformation of old fat body cells into new ones, adapted to the life of an adult insect. The process occurs in the last larval instar. The cells of the larvae are transformed and reduced by peroxisomes, microbodies, mitochondria, and the endoplasmic reticulum. Autophagic and storage vacuoles are constructed. The tissues then dissociate into loose cells, as in the case of histolysis. Immediately after, the cells clump together and organelles are produced [27].

Therefore, the final size of an insect is mainly determined by 20E and IIS, which is decisive for the pace and period of metamorphosis by activating PCD processes and receiving nutritional signals. The influence of IIS and 20E on metamorphosis has been confirmed by 20E activating the AMPK axis and affecting the activation of phosphatase 2 proteins, which cleave phosphate residues from the insulin receptor. This then causes the inhibitory functions of IIS, which inhibits zeroing of *D. melanogaster* [50].

An additional factor influencing the size of the insect and the possibility of proper pupation is the presence of transcription factor hepatocytic nuclear factor 4 in *D. melanogaster* (dHNF4). Recent studies have reported that flies with dHNF4 overexpression have a smaller body size and are unable to pupate [51].

## 4. Role and Metabolism of Lipids

In the cells of the fat body, lipid compounds are stored, released, and processed in the form of droplets and various granules. Fats make >50% of all components in cells and are mainly in the form of anhydrous triglycerides (TGs; 90% of all fats), in combination with a massive water form of glycogen [52,53]. In the hemolymph of insects, lipids can be found in the form of diglycerides (DGs). The accumulated TGs usually exist in the form of drops of fat [14,54]. The drops are made of a chemically inert core composed of TG and cholesterol esters surrounding the core of a monolayer of phospholipids and cholesterol, and peripherally located proteins on a phospholipid layer. The monolayer protects the TGs from unwanted lipolysis due to their low solubility in phospholipids [55]. It is currently unknown how the enzymes enter TGs through the fat droplet layer, but this may be related to the activity of the phosphinothricin acetyltransferase (PAT), lysine-specific histone demethylase (Lsd) 1A (i.e., Lsd1), and Lsd1B (i.e., Lsd2) [14,56]. Droplet proteins, such as perilipin, and several lipases acting on the Lsds [57], take part in the regulation of lipid storage.

Fatty compounds serve as reserves that are mobilized and released as needed to obtain additional energy. The main pathways for the transformation of these compounds are the anabolic lipogenesis and lipolysis pathways [58]. Both pathways are regulated by the appropriate Lsds [57].

**Scheme 2:**
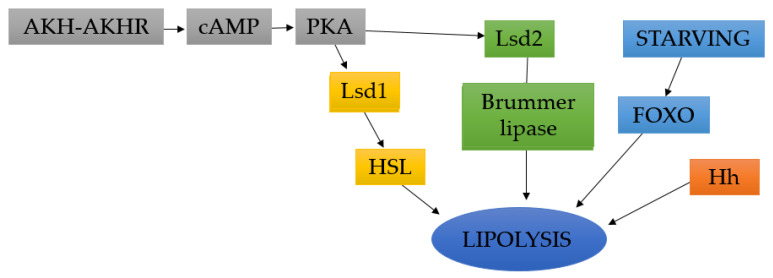
Signals activating lipolysis. AKH–AKHR—adipokinetic hormone—adipokinetic hormone receptor; cAMP—cyclic adenosine monophosphate; PKA—protein kinase A; Lsd 1—lysine-specific histone demethylase 1A; Lsd2—lysine-specific histone demethylase 1B; Hh—hedgehog signaling; HSL—hormone-sensitive lipase; FOXO—Forkhead box-containing transcription factors.

Lipolysis is conditioned primarily by the activity of Lsd1 and Brummer lipase, acid lipase, and hormone-sensitive lipase (HSL). The activation of Brummer lipase is influenced by Lsd2. The activity of Lsd2 and Brummer lipase is due to their phosphorylation by protein kinase A (PKA), which is activated by cyclic adenosine monophosphate (cAMP) production. This, in turn, is regulated by adipokinetic hormone (AKH) and adipokinetic hormone receptor (AKHR). HSL is induced by the activation of Lsd1, which is activated by AKH. In addition, LKB1–AMPK signaling is involved in activating lipolysis. The lipolysis process is inhibited by IIS, which stimulates lipid formation [59,60,61,62,63,64]. Lipolysis can be promoted by nutritional cues such as hunger. Hunger activates Forkhead box-containing transcription factors (FOXO), which inhibits IIS and transactivates Brummer lipase genes and acid lipase [62]. An additional mechanism that activates lipolysis is hedgehog signaling (Hh), which lowers the concentration of TGs and inhibits lipid formation (similar to FOXO) (Scheme 2) [65].

Lipogenesis in *D. melanogaster* is promoted by IIS and the expression of the Sir2, Lipin, and Seipin genes [57,66,67].

The fatty acids present in the fat body act as precursors in the synthesis of eicosanoids, and as hormones that are important for the functioning and development of insects [68]. They are also a component in the synthesis of phospholipids and waxes. Lipophorin is responsible for the transport of lipids in the fat body [58].

### 4.1. Lipogenesis

Starting with lipogenesis, the TGs are the most significant substrate and product of the pathway. The TG, most often found in the hemolymph, very low-density lipoprotein (VDVL), or chylomicrons, is derived from lipoproteins and hydrolyzed by the enzyme lipoprotein lipase. The process is stimulated by the presence of insulin and other VDVL compounds. The products of this hydrolysis are fatty acids and glycerol, components of TGs. The glycerol is then converted into glucose. On the cell surface, acetylCoA synthetase is formed from fatty acids and glycerol-3-phosphate (G3P) from dihydroxyacetone phosphate, which is derived from the transformation of glycerol to glucose with the help of acetylCoA synthetase. G3P can be formed through the following two processes: glycolysis, in which the substrate is glucose, or glycerogenesis from glycogenic substrates (i.e., pyruvate or lactate). Half of the glucose obtained from numerous changes is involved in the synthesis of fats and the stimulation of lipogenesis [14,69].

**Scheme 3:**
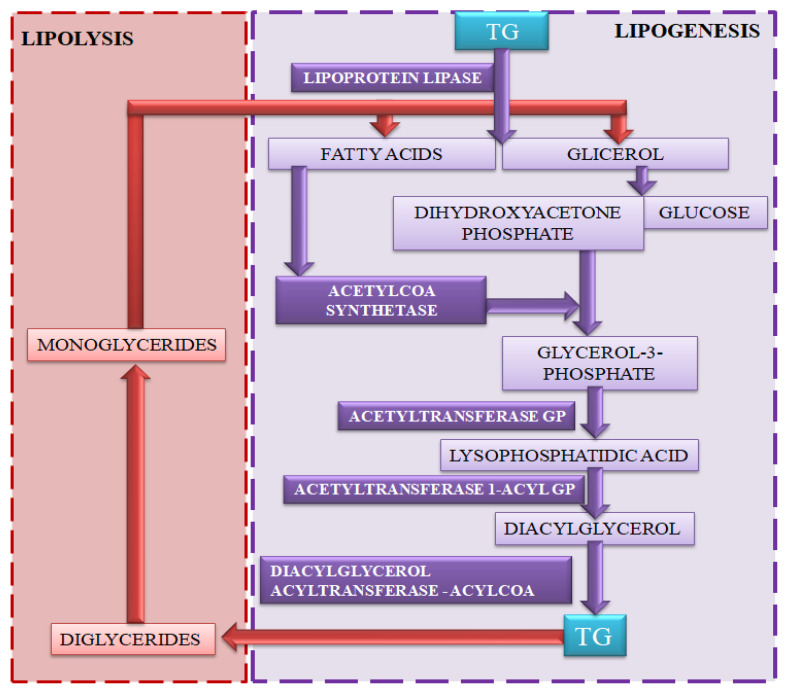
Metabolism of lipids. TG—triglycerides.

In further processes, the end products become the starting substrates again. The G3P obtained from the transformation of TGs undergoes several alcohol esterifications via the following enzymes and processes: (1) acetyltransferase G3P, the product of lysophosphatidic acid; (2) acetyltransferase 1-acyl G3P, the product of diacylglycerol, a DG; and (3) diacylglycerol acyltransferase and acylCoA, re-forming TGs and often constituting a backup form of extreme TG deposition and fat body tissue overgrowth in *Culex pipiens* cells to survive harsh environmental conditions (e.g., starvation). During these processes, phospholipids are by-products that may also be involved in the formation of DGs. DGs can be synthesized by the degradation of phospholipids from phosphatidic acid, from the glycerophosphate pathway, the monoacylglycerol pathway, or lipase-catalyzed triglyceride deacetylation (Scheme 3) [70]. Despite the role of carbohydrates in lipolysis, some insects are unable to convert excess sugars into fats. Such a situation occurs in Hymenoptera and Diptera, despite a diet rich in sugar compounds [14,70,71].

### 4.2. Lipolysis

Lipolysis is the opposite pathway of lipogenesis. The process is based on the constant hydrolysis of TGs to fatty acids. The TGs are first hydrolyzed to DGs and monoglycerides (MGs); as a result of further transformations, the final product is three fatty acid molecules and a glycerol molecule (Figure 2). Due to the poor conversion efficiency of glycerol molecules, some of them end up in the hemolymph of insects. All lipolysis is regulated by the protein perilipine, which prevents or stimulates the hydrolysis/phosphorylation of TGs. Its activity increases during starvation and the action of glucocorticoids, and decreases with food consumption. It stimulates phosphorylation and facilitates its translocation from the cytosol of the cell to the surface of the fat droplets, where TG hydrolysis occurs [14,72].

### 4.3. Lipid Metabolism under Various Environmental Conditions

Lipogenesis is also controlled by other enzymes, such as adipose triglyceride lipase (ATGL), triglyceride lipase (TGL; in *Manduca sexta*), or Brummer’s lipase [14,60]. Lipases are usually hormone-sensitive, which confirms the multidimensionality of these processes. Therefore, lipid transformations (lipogenesis and lipolysis) work differently depending on the stage of development and environmental conditions. This mainly concerns the transformation of glycerides.

In an environment with abundant nutrients, lipids are absorbed from food through the middle intestine and transferred from the intestine to the hemolymph in the form of DGs. At this stage, DGs bind to high-density lipoprotein (HDL; produced from the apoliprotein in the fat body) and then to low-density lipoprotein (LDL). Everything is transported in the body by lipophorin [14].

When consumed, DGs from the hemolymph are bound by LDL and hydrolyzed to fatty acids, which are transported to the fat body tissue. The fatty acids then form TGs in the fat body through the previously described pathways. During increased energy requirements or periods of hunger, DGs are mobilized in the fat body by the action of AKH (Section 8—“Hormons Effect on Fat Body”, page 15). AKH also takes part in the inhibition of 20-hydroxy ecdysone, which inhibits the release of trehalose from fatty acids (Section 5—“Role and Metabolism of Carbohydrates”, page 11) [14].

When used by the ovaries or muscles, DGs are hydrolyzed to fatty acids and transferred to the tissues in need [14,70].

An important aspect of the proper functioning of lipid metabolism is calcium ion homeostasis (Ca^2+^). The proper level of Ca^2+^ is responsible for many functions in the insect organism, including diapause, metamorphosis, hunger sensing, and receiving signals from neurotransmitters. In addition, studies have shown the effect of Ca^2+^ on lipolysis and lipogenesis in invertebrates. The calcium ion level regulates the level of lipid reserves in the fat body (a reduced level in *D. melanogaster* indicates an increase in lipid reserves). In 2021, Dogan et al. investigated the key binding proteins, finding calmodulin, calcineurin, and regucalcin in the *Leptinotarsa decemlineata* fat body. This indicates additional adaptation of this tissue to lipid metabolism [73].

## 5. Role and Metabolism of Carbohydrates

Another energy source is carbohydrates, mainly glucose in the form of the polymer glycogen. Glycogen is an easily activated glucose backup made up of glucose residues. Due to the possibility of its rapid decomposition, insects can quickly release the glucose needed to meet their energy needs [74], as it is often localized in muscle tissues. Glycogen is also found in insect eggs. Glucose, in addition to energy needs, such as movement, is also used to synthesize chitin, which forms the shell of insects [75]. Sugar alcohols formed in metabolism may also help in the adaptation of the body to severe conditions caused by, for example, cold (osmolytes) [16,76]. The transformation of sugars in the insect body uses 35% of the total glucose production (as much as 50% in lipid metabolism) [14,77].

### 5.1. Glycogenic Phosphorolysis

**Scheme 4:**
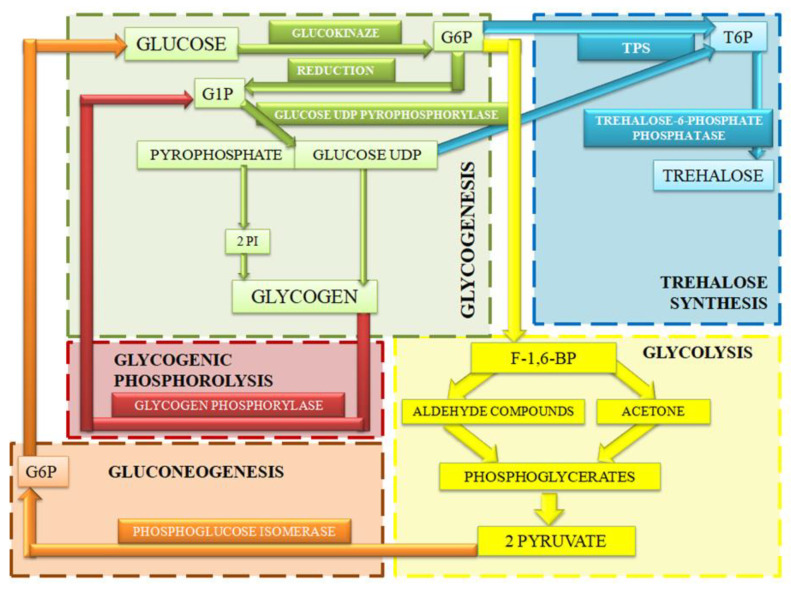
Metabolism of carbohydrates. G6P—glucose-6-phosphate; G1P—glucose-1-phosphate; F-1,6-BP—fructose-1,6-bisphosphate; T6P—trehalose-6-phosphate; TPS—trehalose-6-phosphate synthase.

Glycogen (a spare form of glucose) is converted into glucose-1-phosphate (G1P) by the action of glycogen phosphorylase (GP). G1P is the first substrate in the glycogenesis cycle (Scheme 4). The phosphorolytic cleavage of glycogen, in addition to providing substrates for subsequent reactions, is energetically beneficial because the released sugar is phosphorylated and cannot diffuse out of the cells. GP has very high activity during the intensive growth of the insect (larval phases and the beginning of the imago), when the substrates of this reaction are needed to release glucose for the synthesis of chitin armor. The intensity of phosphorolysis is species-specific [14,77].

### 5.2. Glycogenesis

The conversion of glucose into its glycogen storage form follows the glycogenesis pathway and the insect organism can accumulate supplies for survival in the cells (e.g., diapause or high demand for energy such as during flights). Due to the enzyme glucokinase, the glucose molecule is converted to glucose-6-phosphate (G6P), which is then reduced to G1P. The acting glucose uridine diphosphate (UDP) pyrophosphorylase then converts G1P into UDP glucose and pyrophosphate. The pyrophosphate is converted to two Pi molecules in between (Scheme 4). Due to the resulting UDP glucose molecule, which is the donor of glucose residues, and due to glycogen synthesis, glycogen is maintained with the UDP molecule [14,78,79].

### 5.3. Glycolysis

The task of this cycle is to obtain the greatest possible amount of energy (ATP) from the glucose molecule and supply building components for other reactions. It is the initial stage of the CREBS cycle and the respiratory chain for aerobic organisms. As a result of the glycolysis from one molecule of glucose, we obtain two molecules of pyruvate, which can be used in subsequent reactions (gluconeogenesis), as a result of which we obtain glucose from non-sugar compounds. The glucose is converted successively into G6P, fructose-6-phosphate, fructose-1,6-bisphosphate (F-1,6-BP), acetone or aldehyde compounds, and phosphoglycerates, which eventually form pyruvate molecules (Scheme 4). Pyruvate and other non-sugar compounds are involved in the production of glucose molecules in the gluconeogenesis pathway, which also provides substrates for lipogenesis [18,80,81,82].

### 5.4. Gluconeogenesis

The starting point for this pathway is the pyruvate obtained from glycolysis. During the reaction, other non-sugar compounds may be used, such as lactate (Cori cycle), amino acids, or glycerol, which are pre-converted into pyruvate [83,84]. Pyruvate is converted to G6P by phosphoglucose isomerase, and then GP is hydrolyzed to glucose by an enzyme in the endoplasmic reticulum membrane and directed to the cytosol (Scheme 4) [85].

### 5.5. Trehalose Synthesis

Trehalose is the main disaccharide component of the hemolymph. Sugar, in the form of trehalose, is located in the muscles and used there for high-energy activities, such as flying. Interestingly, bees that are resistant to microsporidia *Nosema* spp. demonstrate a higher level of trehalose in their cells [17,18,20]. Trehalose is synthesized from trehalose-6-phosphate (T6P), which is formed from UDP glucose and G6P in the presence of trehalose-6-phosphate synthase (TPS). The resulting trehalose-6-phosphate is then dephosphorylated by trehalose-6-phosphate phosphatase (TPP) into the free trehalose molecule (Scheme 4) [86]. Trehalose is a very important compound produced during the sugar cycles. This has been suggested by studies that have found that the priority in some insects is the synthesis of trehalose from glucose. Only when the level of trehalose reaches an appropriate level does its synthesis stop and glycogen synthesis begin [87]. In mosquitoes and locusts, the synthesis of trehalose is a priority over the synthesis of lipids. In some insects, trehalose undergoes a further transformation. For example, in Colorado beetles, trehalose is transformed into proline and used [88]. In addition, trehalose reduces the lipid concentration in the hemolymph, due to its inhibitory effect on lipolytic activity (influence on the rate of lipolysis) by reducing the activity of the lipase enzyme [20,87].

## 6. Role and Metabolism of Proteins

Proteins and amino acids in the body of insects (e.g., proline, arylforin) perform a similar function to other organic compounds. They are an energy source, a substrate for other sugar transformations, and building blocks for other molecules. Protein reserves are usually used during metamorphosis, when the insect uses previously accumulated resources. The rate of growth and reproduction time depend on the amount of resources due to the presence of reserve sensors (expression of amino acid transporters), which determine the right moment of growth/metamorphosis/molting at the time of adequate nutrient accumulation. Protein metabolism is controlled by 20E and JH, the same hormones as in metamorphosis. During the larval stage, the protein undergoes constant synthesis, but the maximum yield of synthesis is in the last larval stage, before metamorphosis. Intensely accumulated proteins help the insect survive the period without food [18,27,89]. In addition, the accumulated proteins are used to build a new imago body. During and immediately after metamorphosis, the protein content decreases. Proteolysis is activated by FOXO (cotton bollworm). Protein transformations also include the urea cycle and the detoxification of the metabolites in the nitrogen pathway. Glycogen and amino acids, such as proline, may be the initial substrate in the gluconeogenesis pathway (the use of non-sugar compounds to produce glucose) [18,27,37,89].

### 6.1. Urea Cycle

Urea/fatty acids are held in granules or vacuoles located in the cells of the fat body [90,91,92]. This and other functions of this tissue suggest that the fat body may play the role of the liver in insects. During the development of the insect, when the Malpighian tubule system (MT) is made, the accumulated urea is released through the tubules to the outside [93,94]. This is confirmed by the presence of white frost from urea acid on the surface of the *M. sexta* hairs immediately after the formation of the MT. This hypothesis can also be confirmed by the reduction in urea accumulation in storage vacuoles (before transformation, urea constitutes about 75% of the vacuole content) and the reduction in its volume in an adult insect [27].

In the urea cycle, ammonia is converted to its less toxic version, urea. Due to the presence of xanthine dehydrogenase, urea is converted into urea acid and excreted from the body [27,95].

### 6.2. Proline

For some insects, proline is the form of amino acid used most. Proline is synthesized in the cells of the fat body from acetylCoA and alanine. During the synthesis of proline, the concentration of alanine increases. The proline that is formed is transported from the fat body to the hemolymph. Its use is possible due to the participation of muscle mitochondria, where this compound has the greatest application [90]. There is a digestive enzyme in the mitochondria that oxidizes proline, as well as pyruvate and fatty acids. Proline is used as the main source of energy in the tsetse fly [96].

### 6.3. Protein Sensors

Taking food or not activates the appropriate pathways at a given time, affecting the economy of the whole organism. Reducing the amino acid intake and RNA expression, and knocking down amino acid transporter genes, reduces the activity of TORC1 and the production of fat body signals (FBSs), which control the production of insulin-like peptides (ILPs) from insulin-producing cells (IPCs) in the insect brain, and promote the production of ILP6 in the brain glial cells, which enables neuroblast rest. The production of ILP from IPCs is promoted by the activity of the Methuselak receptor present in the IPCs [97,98,99].

## 7. Additional Metabolic Sensors

At the time of starvation, the Janus kinase (JAK) activates the Grindelwald receptor (Gmd) in the IPCs. After its activation, ILP production from IPCs is inhibited [46].

ILP is also produced when the tissue senses the presence of glucose. After a meal, the tissue responds to the supplied nutrients by activating the FBS peptides. The peptides promote unpaired 2 (Upd2) or CCHamide-2 (CCHa2). The promotion of Upd2 and CCHa2 induces the production of ILP (lectin homolog), which then activates the JAK/signal transducer and the activation of transcription (STAT). JAK/STAT activates the neurotransmitter gamma-aminobutyric acid (GABA) and ultimately promotes ILP by guiding the signals to the neurons [63,100].

The state that is the opposite of hunger is satiety. Female-specific independent transformer (FIT) is present in females, which is an indicator of the organism’s fulfillment of nutritional needs. FIT promotes the secretion of ILP2, which limits protein intake relative to the sexual dimorphism of insects [101].

## 8. Hormone Effect on Fat Body

In addition to numerous compounds, enzymes, and substances, metabolism and other processes are influenced by the presence of hormones regulating the activity of changes inside the tissue of the fat body. The development and moment of molting or the transition of metamorphosis in insects also depend on the action of hormones. The basic hormones most often regulating processes are AKH, ecdysteroid (Ecd), JH, ILPs, diapause hormone–pheromone biosynthesis-activating neuropeptide (DH-PBAN), corazonin (CRz), leucokin (Lk), CCHa2, allanostatin-A (Ast-A), tahykinin (Tk), limostatin (Lst), cytokines, short neuropeptide F (sNPF), and neuropeptide F (NPF) [14,18,102,103,104].

AKH is a peptide produced by the cardiac corpora neurosecretory cells. AKH is also expressed in the ovaries, midgut, fat body, and muscle tissues. It consists of 8–10 amino acids and is similar to glucagon [13]. Many insects have several AKHs, and migratory locusts have three variants, which exhibit different bioactivity from others [105]. The hormone initially exists in the form of a prohormone that, when activated, is released by the cleavage of AKH from the adipokinetic hormone precursor-related peptide (APRP). AKH activity is present in the most important stages of development, due to the regulation of energy reserves and their mobilization in the body of insects during molting and metamorphosis [103]. It works mainly by stimulating the work of enzymes, such as glycogen phosphorylase (conversion of sugars from glycogen) and triglyceride lipase (during lipid metabolism) (Section 4—“Role and Metabolism of Lipids”, page 8). AKH production is often a response to rapid changes in lipid levels. The hormone is mobilized by the AKHR transduction signal (i.e., AKHR). The AKHR then influences the activation of phospholipase C, which cleaves the membrane lipids into inocytol-1,4,5-triphosphate and diacylglycerol. At the same time, AKHR affects inositol trisphosphate (IP3), which causes an increase in calcium ions in the endoplasmic reticulum and transfer to the cytosol. In addition, the activation of the hormone influences the initiation of the action of cyclase adenylases, and then the production of cAMP [106]. Thus, AKH activation regulates the level of TGs in the fat body [107]. The inhibition of the glycolysis pathway by the action of AKH occurs as a result of a decrease in the supply of a substrate for this reaction (fructose-2,6-bisphosphate) and a decrease in the activity of phosphofructokinase. AKH also influences the rate of hydrolysis of TGs and their release into the hemolymph in the form of DGs [14,102]. The hormone also acts on CREB, calcium homeostasis, and the expression of fatty degeneration genes; stimulates heartbeat, locomotion, and neuronal signaling; increases muscle tone; and protects against oxidative stress [103,108,109,110]. 

JH is responsible for many processes related to the development time of the larva and imago, but is also responsible for stimulating the synthesis of vitellogenin, an important precursor of the yolk protein taken into the oocytes. The presence of JH has been shown to control the presence of protein granules, and the lack of this hormone is a signal for metamorphosis by stimulating cytolysis of the larval fat body and the synthesis of a new one. In addition, the JH-1 analogue causes vacuolization of old trophocytes. The presence of this hormone at a low concentration stimulates the production of vitallogen in the fat body (i.e., vitellogenin) [18,102,111].

Ecd simultaneously works with and inversely to JH. Ecd regulates the time of metamorphosis by stimulating tissue dissociation (metamorphosis stage), tissue remodeling, and the appearance of autophagous structures. JH inhibits premature metamorphosis and aging. A lack of any of these hormones causes severe malformations, molting problems, and failure to metamorphose. Incorrect molting time and date affect the development and maximum use of nutrients in building the appropriate weight and anatomical development [102].

ILPs are peptides produced in IPCs, located in the medial and lateral parts of the nervous system of the brain and heart. Production also occurs in salivary glands (ILP6), ovaries, Malpighian tubules (ILP5), larval midgut, and mesoderm (ILP2). This group of hormones has a controlling and regulating effect on carbohydrate levels [112]. As is the case with many hormones, several variants have been distinguished in this group for a given insect; for example, in *D. melanogaster* there are eight of them (DILP1-8) [113,114]. ILPs act mainly on lipid transformations in lipogenesis and lipolysis. ILPs will inhibit FOXO signaling, activate sterol regulation, and control lipid accumulation during diapause. ILP6 is also responsible for the suppression of ILPs and increased resistance to hunger [103].

DH-PBAN has been recorded mainly in *B. mori* and *Helicoverpa armigera*. It is produced by neurosecretory cells in the subesophageal ganglion. Its main purpose is to regulate diapause in insects. In silkworms, DH-PBAN induces diapause, whereas in *H. armigera* it acts to reactivate metabolism after the insect’s resting state. The action of the peptide is possible due to the activation of the PBAN gene, which codes for DH. PBAN is also involved in the biosynthesis of female sex pheromones [115].

CRz is produced by the brain’s neurosecretory cells. The hormone is evolutionarily related to AKH. It mainly affects social behavior, behavior and caste identification (also reproductive behavior and sperm transfer), melanization, and stress reaction [116,117,118,119,120]. Changes in CRz expression reduce the level of trehalose and affect the concentration of lipids and carbohydrates (increased TG content and acceleration of glucose circulation in the hemolymph).

Lk is a myotropic peptide produced by the brain, ventral ganglia, and IPL-producing cells. The main task is to maintain the homeostasis of ions and water in the body by regulating the secretion of fluids from the Malpighian tubules, regulating behavior and physiology before and after eating, and the feeling of hunger itself [103].

The hormone/peptide CCHa2 is produced in the fat body and intestine [121]. It reduces food activity, which may affect the time (late) of pupation in individuals with the CCHa2 mutation [100]. The CCHa2 regulation of ILP growth and the induced larval growth time also influence the turnover time [103].

Ast-A is produced by the brain and intestine. Its activity affects the regulation of other hormones, specifically AKH and ILPs [122]. Its role is to regulate nutrient homeostasis and lipid accumulation by sex and diet. Ast-A expression is different in males and females [103].

Tk is a hormone produced in the central nervous system and the intestine. Its expression affects the metabolism and storage of lipids, regulating TGs and insulin signaling. Tk deficiencies cause lipogenesis, increasing the intestinal TG content. It also affects the intestinal immunity [103].

Lst is produced by AKH-producing neurons in the corpora cardiac. Hormones suppress the production of insulin during starvation (i.e., regulate ILP and AKH) and regulate the increase in lipolysis [103].

Cytokines are mainly secreted in the cells of the fat body (Upd2) and the brain (Upd1). These protein hormones are similar to lectins. Upd2 acts on IPCs and their release of ILP, and the activation of insulin signals. Moreover, they regulate GABA [103].

Further, sNPF and NPF are hormones that inhibit peristalsis and the release of digestive enzymes, and preserve nutrition. They are mainly produced by the brain, but also in the middle intestine, large intestine, antennae, and Malpighian tubules [103]. Gene silencing results in reduced food consumption and the development of lean individuals in flies, whereas its overexpression may cause fat deposition [103].

## 9. Role in Immunity

In addition to the transformation of organic compounds, most of the immune proteins present in the hemolymph are synthesized in the fat body. Protein synthesis plays a role in the innate humoral (innate level of immune proteins, e.g., lysozyme) and acquired immune responses (synthesis of antibacterial proteins, e.g., attacin, defensins, etc.) [14,123,124]. In insects that have previously been in contact with the pathogen, the production of immune proteins and polypeptides in the fat body is stimulated by hemokines, which act as transmitters between the environmental hemolymph and the fat body. When infection of the body cavities occurs, effectors are produced after a few hours and transferred to the hemolymph [123,125,126]. Notably, most of the information on the functioning of the immune system of insects has been obtained by tests on the order Diptera [127].

Unwanted microorganisms/substances/cell structures rendered harmless by peptides are transported to the fat body due to phagocytosis led by hemocytes in the hemolymph [128].

**Scheme 5:**
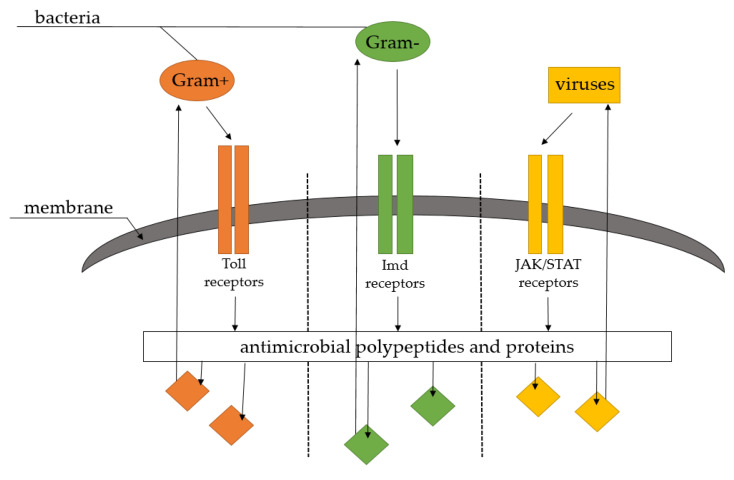
Mechanism of action of immune pathways. Toll—proper name of Toll group receptors; Imd—name from immune deficiency”; JAK/STAT—janus kinases/signal transducers and activator of transcription activities.

Microbial recognition and defense activation relies on the recognition of pathogen-associated molecular patterns (PAMPs) by pattern-recognition receptors (PRRs). Depending on the type of pattern, which varies for Gram-positive and Gram-negative bacteria, Toll, Imd, or JAK/STAT signaling is activated for the induction of antimicrobial peptide synthesis. Toll is activated after the entry of Gram-positive bacteria into the body and Imd after the penetration of Gram-negative bacteria. The identification of PRRs occurs due to the recognition of the components of the bacterial cell wall, i.e., peptidoglycans (i.e., peptidoglycan recognition proteins (PGRPs) or beta-1,3-glucans type C lectin). JAK/STAT and RNAi participate in the fight against viruses (Scheme 5). The activation of Toll, JAK/STAT, and Imd also regulates cytokines, reactive oxygen species (ROS), nitrogen oxides, and biogenic amines [129,130,131,132,133,134,135].

Invertebrate organisms also have specialized structures to fight fungi. Metalloprotease inhibitors are designed to neutralize proteases secreted by fungi that destroy invertebrate immune proteins. A significant effect on the resistance of these inhibitors was demonstrated in a study involving *Galleria mallonella*, by inactivating the IMPI gene responsible for the production of metalloprotease inhibitors, which made the insects more susceptible to infection with *Metarhizium brunneum* ARSEF4556 [136].

The greatest synthesis occurs in the larval stages. After transplantation, the synthesis decreases and the previously accumulated proteins are stored in granules. Their synthesis is initiated by beta-exdysone under the control of the endocrine system. The most important antimicrobial proteins are cecropins (Lepidoptera), attacins (Lepidoptera), apidicins, abycin (Hymenoptera, Apidae), diptericins (Diptera), and lysozyme or lectins. Some of them are described below [123].

### 9.1. Lysozyme

In insects, lysozyme is an enzyme with bacteriolytic properties. It is a low-molecular-weight alkaline protein that catalyzes the hydrolysis of bonds present in the cell walls of bacterial cells. The lysis of the bacterial cell wall allows the hemolymph, with proteases, hemokines, and other chelating substances, to enter its interior. The advantage of lysozyme is that it attacks sensitive bacteria, usually Gram-positive bacteria, and is saprophytic in various environments. In addition, in order for the action of lysozyme to be visible in the bacterial envelope, murein must be present. Its activity has been detected in aerobic and anaerobic conditions, as well as acidic and alkaline environments. In an uninfected insect, the level of lysozyme is 5–1000 qg/mL of hemolymph [123]. The initial concentration depends on the type of insect and its shell structure. In the Hymenoptera (Apidae) insects, the innate activity of lysozyme is very low, if even traceable. In Lepidoptera, the level of lysozyme is higher than in Hymenoptera, but it varies individually. The highest level of lysozyme is observed in insects of the orders Diptera, Blattidae, and Isoptera. As a rule, insects with thinner armor produce greater amounts of lysozyme due to the greater risk of damage and penetration of a pathogen into the body cavities. The activity of lysozyme also has a stimulating effect on the hemocyte response and can interact with other proteins, such as apidicin and abycin, helping to completely destroy the bacterial cell membrane [123,137,138].

### 9.2. Lectins

Lectins are proteins found in most insects, usually in the larval stage. Their task is to recognize foreign substances in the body via the attachment of their structure to bacterial cell surface polysaccharides (e.g., the M13 protein found in *M. sexta* and *A. mellifera*). Lectins, otherwise known as agglutinins, cause aggregation of unwanted cells, allowing them to be removed from the body faster by encapsulation (Sarcophaga lectin). In addition, lectin proteins support metamorphosis in insects by facilitating phagocytosis (the remains of old cells are removed) [123].

### 9.3. Cecropins

Cecropins have a much broader range of antibacterial activity. Compared to other proteins and polypeptides, they are active against both Gram-positive and Gram-negative bacteria. Cecropins are active against *Pseudomonas areoginosa*, *Serratia marcescens*, and *Xenorhabdus nematophilus*. They occur in most holometabolic insects in the form of a basic polypeptide (in Lepidoptera). Cecropin molecules are synthesized through several processes. When a pathogen enters a body cavity, the cells of the hemolymph capture foreign cells and phagocytose them. The signal can be induced not only by bacterial cells, but also by dead cells or substances. The hemocytes filled with such phagocytes activate the synthesis of specific mRNA in the fat body. Specific mRNA appears in the tissue, which then stimulates the fat body to synthesize the target cecropins. There are several types of cecropins, but the most active are cecropins type A and B. Some proteins (e.g., Sarcophaga cecropins) bind to the cell membrane of prokaryotes, destroy liposomes, and induce the formation of ion channels. They also have the ability to block proline use and cause ATP loss. The complete destruction of bacterial cells requires cooperation between cecropins and lysozyme, to remove the remnants of the cell wall. Proteins with cecropin activity include sacrotoxin I (*Sarcophaga perigrina*) and andropin (*Drosophilia*) [123,139].

### 9.4. Attacin

Other antimicrobial proteins are the numerous Lepidoptera attacins present in the insect body. Attacins are composed mostly of aspartic acid, glycine, alanine, phenylalanine, and threonine and, depending on the amino acid sequence, they can be of six types (basic and acidic forms). Proteins with attacin activity are mainly responsible for protection against *Pseudomonas manthophilia*, *Escherichia coli*, and *Acinetobacter colcoaceticus*, affecting the permeability of the cell membrane during the division phases. Other proteins with attacin activity include diptericins (in Diptera), sacrotoxins II and III (*Sarcophaga perigrina*), and tin coleopteria (Coleoptera) [102,123].

### 9.5. Defensin

The activity of the polypeptide defensin depends on the amount produced. Defensin works well against Gram-negative bacteria at any concentration. In order to be effective against Gram-negative bacteria, more is needed. As in the case of other proteins or polypeptides, defensins attach to the membrane of bacterial cells with a specific composition, which a given defensin is able to break down (i.e., sapecins have an effect on cell membranes containing cardiolipin). One of the defenses that is produced in the fat body is formicin extracted from *Phormia terranovae* [123,140].

### 9.6. Apidicin and Abycin in Apidae Insects

Hymenoptera insects, the bees, produce specific antibacterial proteins and polypeptides. Apidicin is active against pathogens widely present in the environment of bees. These are most often phytopathogens found on plants and inhabiting the digestive tract of humans and animals. This allows for the control of unwanted microorganisms, such as during harvesting by workers. The presence of apidicin is considered to be one of the adaptation processes of bees’ immunity to their environment. Both abycin and apidicin are rich in proline (30% of the protein). Abicin reacts most strongly to *E. coli* infections, but the most sensitive bacterium to its action is *Xantohomonas campestris*. The second polypeptide in bees is more active against Gram-positive bacteria than Gram-negative bacteria, with weaker and delayed action compared to apidicin [124,141].

### 9.7. Reaction to Toxins

In addition to microbes, insects have also faced the problem of the toxicity of substances present in their environment. As a result, immune processes have been recognized that are helpful in defending against such a threat. Initially, cytochrome P450 monooxygenase, esterases, and flavin monoxygenases [142] were shown to reduce the toxicity of biologically active substances (i.e., xenobiotics, endogenous substances). The toxic products of metabolism are protected by glutathione-S-transferase (GST) and UDP gluconosyl transferase (UGT) [143,144]. Eventually, the toxins are transported from the cells by transporters and ATP-binding cassettes (ABCs) [142]. Resistance to xenobiotics is determined by the expression of a gene from the CYP3 family [145,146].

### 9.8. Vitellogenin

An important aspect for the insect organism is the synthesis of yolk protein precursors (e.g., vitellogenin) in the fat body. The majority of this protein is found in sexually mature and breeding females. During development, the oocytes themselves synthesize vitellogenin, which is needed in the first stages of development, but after some time they need to extract an additional amount from the hemolymph. The protein synthesized in the body is sent to the hemolymph and used by the ovaries and the egg. The cells that produce this protein are rich in rough endoplasmic reticulum, with numerous ribosomes. The appropriate production and uptake of vitellogenin determines the proper development of the oocyte, and the subsequent development of the egg larvae [147,148,149].

### 9.9. Immunosenescence

Immunosenescence refers to the aging of the body with and through the aging of key immune tissues. With age, tissues change and deregulate; as a result, a generalized inflammation may appear in the body caused by the existing diseases related to aging and the formation of tumors. Changes in the functioning of tissues have a genetic basis. In the fat body, the expression of the IIS-TORC1 pathway genes involved in metabolism decreases over time. In addition, the gradual loss of B-lamin triggers induced stem cells. Chronic inflammation causes continuous sterile Toll activation. All of these signals shorten life. During inflammation, the main signaling molecules are eicosanoids, which are also associated with the prostaglandin receptor (PGE2) [150,151,152]. Despite mediating many processes in vertebrates, prostaglandin may have a negative effect in the case of invertebrate immunity. After the use of prostaglandin I_2_ by Ahmed et al., they noted immunity suppression and a problem in oocyte development and reduced fertility in *Spodoptera exigua* [153].

## 10. Conclusions

The fat body is one of the most important tissues in the body of insects due to the number of functions that its cells perform. The metabolism of nutrients allows for proper growth and development, and the use of the immune potential against the external environment. The fat body tissue integrates many signals from many pathways that receive and/or transmit to the hemolymph, which affects the functioning of the entire body. The transformation of lipids, carbohydrates and proteins allows insects to survive in harsh conditions or during diapause, due to the accumulated reserves and their quick release during energy demand. The reserves allow the survival of insects that, in adulthood, did not develop a mouth apparatus capable of feeding (e.g., *B. mori*). Many proteins and antibacterial polypeptides are synthesized in the tissues of the fat body, thanks to which the body has a chance to defend itself against microorganisms penetrating through anatomical barriers into the body cavities. The fat body is responsible for the innate and acquired humoral defense. Proteins and polypeptides, including lysozyme, cecropins, attacins, and defensins, exhibit a wide spectrum of activity against microorganisms and, with the cooperation with hemocytes, insects are able to phagocytose and remove them from their body. In addition, the proteins necessary for the development of oocytes are synthesized in the fat body, allowing the larva to develop from the egg. Many functions of this tissue resemble the liver, to which it is often compared. Despite the availability of a lot of information on the structure and biochemistry of the fat body, it is an interesting research topic for which a lot is still to be discovered.

## Abbreviations

20E	20-hydroxyecdysone
93F	proper name of protein
ABC	ATP binding cassette
AKH-AKHR	adipokinetic hormone—adipokinetic hormone receptor
AMPK	5′ adenosine monophosphate-activated protein kinase
APRP	adipokinetic hormone precursor-related peptide
Ast-A	allanostatin-A
ATG	autophagy-related proteins
ATGL	adipose triglyceride lipase
Br-C	Broad-Complex
CAC	caspase activity cascade
cAMP	cyclic adenosine monophosphate
CCha2	CCHamide-2
Ckd2	cyclin-dependent kinase 2
Ckd4	cyclin-dependent kinase 4
CRz	co-razonin
DG	diglyceride
DH-BPAN	diapause hormone-pheromone biosynthesis activating neuropeptide
DILP1-8	insulin-like peptides in *Drosophilia melanogaster*
E93	ecdysone-induced protein 93
Ecd	ecdysteroid
EcR/USP	ecdysone receptor/ultraspiracle protein
F-1,6-BP	fructose-1,6-bisphosphate
FBS	fat body signal
FIT	female-specific independent transformer
FOXO	Forkhead box-containing transcription factors
G1P	glucose-1-phosphate
G3P	glycerol-3-phosphate
G6P	glucose-6-phosphate
GABA	gamma-aminobutyric acid
Gmd	Grindelwald receptor
GP	glycogen phosphorylase
GST	glutathione-S-transferase
HDL	high-density lipoprotein
Hh	hedgehog signaling
HSL	hormone-sensitive lipase
IIS	insulin/IGF signaling
ILP6	insulin-like peptide 6
ILP	insulin-like peptide
IP3	inositol trisphosphate
IPC	insulin producing cell
JAK/STAT	Janus kinase/signal transducers and activator of transcriptio
JH	juvenile hormone
Kr-h1	Krüppel homolog
LDL	low-density lipoprotein
Lk	leucokin
LKB1	LKB1 kinase
Lsd1	lysine-specific histone demethylase 1A
Lsd2	lysine-specific histone demethylase 1B
Lst	limostatin
MG	monoglyceride
Mmps	metalloproteinase
MT	Malpighian tubule system
Myc	proper name of gene regulator
NPF	neuropeptide F
PAMP	pathogen-associated molecular pattern
PAT	phosphinothricin acetyltransferase
PCD	programmed cell death
PGE 2	prostaglandin receptor
PGRP	peptidoglycan recognition protein
PKA	protein kinase A
PRR	pattern recognition receptor
ROS	reactive oxygen species
Sir2	sirtuin homolog
sNPF	short neuropeptide F
T6P	trehalose-6-phosphate
TG	triglyceride
TGL	triglyceride lipase
Tk	tahykinin
Toll	proper name of Toll group receptors
TORC1	transducer of regulated CREB activity 1
TPP	trehalose-6-phosphate phosphatase
TPS	trehalose-6-phosphate synthase
UDP glucose	uridine diphosphate glucose
UGT	UDP gluconosyl transferase
Upd2	unpaired 2
VDVL	very low-density lipoprotein
